# Hexosamine Biosynthetic Pathway-Derived O-GlcNAcylation Is Critical for RANKL-Mediated Osteoclast Differentiation

**DOI:** 10.3390/ijms22168888

**Published:** 2021-08-18

**Authors:** Myoung Jun Kim, Hyuk Soon Kim, Sangyong Lee, Keun Young Min, Wahn Soo Choi, Jueng Soo You

**Affiliations:** 1 School of Medicine, Konkuk University, Seoul 05029, Korea; wns798@naver.com (M.J.K.); sangyong@konkuk.ac.kr (S.L.); m.m1203@hanmail.net (K.Y.M.); wahnchoi@kku.ac.kr (W.S.C.); 2 Department of Biomedical Sciences, College of Natural Science, Dong-A University, Busan 49315, Korea; hskimxo@dau.ac.kr; 3 Department of Health Sciences, The Graduate School of Dong-A University, Busan 49315, Korea; 4 KU Open Innovation Center, Research Institute of Medical Science, Konkuk University, Chungju 27478, Korea

**Keywords:** O-GlcNAc transferase, O-GlcNAcylation, osteoclastogenesis, osteoclast, NFATc1, p65

## Abstract

O-linked-N-acetylglucosaminylation (O-GlcNAcylation) performed by O-GlcNAc transferase (OGT) is a nutrient-responsive post-translational modification (PTM) via the hexosamine biosynthetic pathway (HBP). Various transcription factors (TFs) are O-GlcNAcylated, affecting their activities and significantly contributing to cellular processes ranging from survival to cellular differentiation. Given the pleiotropic functions of O-GlcNAc modification, it has been studied in various fields; however, the role of O-GlcNAcylation during osteoclast differentiation remains to be explored. Kinetic transcriptome analysis during receptor activator of nuclear factor-kappaB (NF-κB) ligand (RANKL)-mediated osteoclast differentiation revealed that the nexus of major nutrient metabolism, HBP was critical for this process. We observed that the critical genes related to HBP activation, including *Nagk*, *Gfpt1*, and *Ogt*, were upregulated, while the global O-GlcNAcylation was increased concomitantly during osteoclast differentiation. The O-GlcNAcylation inhibition by the small-molecule inhibitor OSMI-1 reduced osteoclast differentiation in vitro and in vivo by disrupting the translocation of NF-κB p65 and nuclear factor of activated T cells c1 (NFATc1) into the nucleus by controlling their PTM O-GlcNAcylation. Furthermore, OSMI-1 had a synergistic effect with bone target therapy on osteoclastogenesis. Lastly, knocking down *Ogt* with shRNA (*shOgt*) mimicked OSMI-1’s effect on osteoclastogenesis. Targeting O-GlcNAcylation during osteoclast differentiation may be a valuable therapeutic approach for osteoclast-activated bone diseases.

## 1. Introduction

During cellular differentiation, cells acquire specific identities in response to environmental cues or cellular signals. Cells use various strategies, from translational regulation to post-translational modification, to appropriately respond to stimuli. Post-translational modification (PTM) refers to the covalent and enzymatic modification of proteins following protein biosynthesis [1]. O-linked-N-acetylglucosaminylation (O-GlcNAcylation), the attachment of O-linked N-acetylglucosamine, is one of the emerging PTMs found on the serine and threonine residues of nucleocytoplasmic proteins [2]. O-GlcNAcylation is a nutrient sensor through the hexosamine biosynthetic pathway (HBP) [3,4]. HBP is responsible for producing uridine diphosphate-N-acetylglucosamine (UDP-GlcNAc), a key substrate for protein O-GlcNAcylation. HBP utilizes molecules from the metabolism of carbohydrates (glucose), lipids (acetyl-CoA), amino acids (glutamine), and nucleotides (uridine triphosphate) to generate UDP-GlcNAc [5]. O-GlcNAcylation couples the fluctuations in nutrient availability with downstream signaling pathways; thus, O-GlcNAcylation homeostasis is physiologically and pathologically consequential [5,6,7,8,9]. 

Research on the functions of O-GlcNAcylation is ongoing. O-GlcNAcylation affects protein stability and protein–protein interactions [10]; however, its downstream effects are much more complicated. Interestingly, unlike other PTMs, these O-GlcNAcylations are controlled by a single pair of enzymes, O-GlcNAc transferase (OGT) and O-GlcNAcase (OGA). OGT catalyzes the transfer of a GlcNAc moiety from UDP-GlcNAc, the donor substrate, to a serine or threonine residue in proteins. Conversely, OGA, which catalyzes the hydrolysis of the sugar modification, removes the O-GlcNAc [2]. Although the OGT and OGA genes are both expressed in the nucleus and cytoplasm, OGT is predominantly present in the nucleus and OGA is enriched in the cytoplasm [11]. The O-GlcNAcylation of the master transcription factors (TFs) has been reported in various biological systems, including keratinocytes [12], liver cells [13], B cells [14], T cells [14,15], adipose tissues [16,17], and neurons [18,19]. For instance, the O-GlcNAcylation of NOTCH proteins, T cell antigen receptors, and c-MYC in T cells are critical for T cell self-renewal and malignancy [20]. O-GlcNAcylation can affect TF activity via a couple of mechanisms, such as protein translocation and stability. Interestingly, one TF, nuclear factor-kappaB (NF-κB), is regulated by different O-GlcNAcylations simultaneously. The O-GlcNAcylation of its RelA subunit interferes with its binding to NF-κB inhibitor alpha (IκBα), enhancing its nuclear translocation and transcriptional activity. Meanwhile, the O-GlcNAcylation of its c-Rel subunit is essential for NF-κB’s DNA binding and transactivation activity [14,15,21]. There have been reports on the O-GlcNAcylation of major TF produced during cell fate determination, such as cell differentiation [12,14,16,18,22,23]; however, it remains unclear how OGT and OGA recognize numerous substrates and control downstream signals and how O-GlcNAcylation coordinates the entire molecular network in specific cellular contexts.

Bone is a dynamic tissue that maintains homeostasis via various factors, including cytokines, chemokines, hormones, and mechanical stimuli [24,25]. Bone homeostasis is controlled by bone formation and bone resorption. The processes regulated by osteoclasts and osteoblasts continue to resorb and regenerate the bone tissue [26]. Osteoclast differentiation is a highly dynamic process. As with the cells in other differentiation processes, osteoclasts are susceptible to subtle changes in the external environment. The role of O-GlcNAcylation in osteoblast differentiation remains controversial. Some studies have reported that osteogenesis is positively regulated by O-GlcNAcylation [27,28,29,30]. The master TF of osteoblast differentiation, Runt-related transcription factor 2 (Runx2), is O-GlcNAcylated and able to control alkaline phosphatase, an early marker of bone formation. Conversely, a recent study reported that increased O-GlcNAcylation of Runx2 reduces transcriptional activity and induces an imbalance in bone homeostasis in hyperglycemia [31]. Osteoblast differentiation is inhibited by excessive O-GlcNAcylation induced by hyperglycemia. The connection between glucose metabolism and bone-related diseases has been extensively studied [32,33,34,35]; however, HBP, a branch of glycolysis, has only recently begun to be investigated.

In this study, we observed that HBP was activated and global O-GlcNAcylation was increased during murine bone marrow-derived osteoclast differentiation by analyzing a kinetic transcriptome. Then, we investigated the role of O-GlcNAcylation in osteoclastogenesis.

## 2. Results

### 2.1. HBP Is Activated and O-GlcNAcylation Is Increased during RANKL-Mediated Osteoclast Differentiation

**Figure 1:**
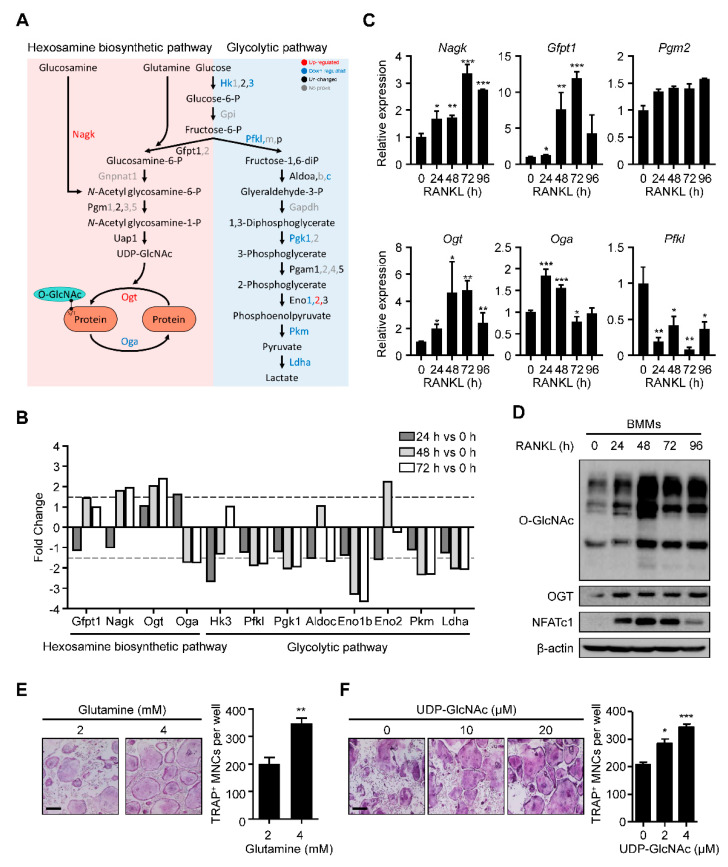
O-GlcNAcylation is required for RANKL-mediated osteoclast differentiation. (**A**) The transciptome data (GSE176265) showed relative mRNA expression levels of the glycolytic pathway and HBP during osteoclast differentiation. (**B**) The fold changes of the HBP and glycolytic pathway genes are illustrated on a graph. Dotted lines represent 1.5-fold differences. (**C**) Relative mRNA expression levels of HBP during osteoclast differentiation, as measured by RT-qPCR. (**D**) The RANKL-induced global O-GlcNAcylated protein and Ogt protein levels, as confirmed by Western blot analysis. (**E**) High amounts of glutamine (4 mM) increased the TRAP-positive multinuclear cells compared with low amounts of glutamine (2 mM). The TRAP-specific mature osteoclasts were counted (left panel). Scale bar, 200 μm. (**F**) High amounts of UDP-GlcNAc, the end product of HBP, increased the TRAP-specific mature osteoclasts compared with the control culture. The TRAP-specific mature osteoclasts were counted (left panel). Scale bar, 200 μm. Statistical data are presented as the means ± S.E.M. (* *p* < 0.05, ** *p* < 0.01 and *** *p* < 0.001, as measured by Student’s *t*-test).

We explored the molecular metabolism of osteoclast differentiation by first checking the HBP-related gene expression. We used our previous kinetic transcriptome upon RANKL stimulation (GSE176265). While the levels of most glycolytic pathway enzymes were downregulated, there was substantial upregulation of N-acetylglucosamine kinase (Nagk) and Ogt and downregulation of phosphofructokinase, liver type (Pfkl) and Oga (Figure 1A,B). Real-time PCR confirmed the significant upregulation of Nagk and Ogt, although downregulated genes revealed the pattern with low significance (Figure 1C). Additionally, glutamine–fructose-6-phosphate aminotransferase 1 (Gfpt1), whose enzyme increased glucose flux into HBP, and phosphoglucomutase 2 (Pgm2) were also observed to be upregulated, although their changes were not as significant in the transcriptome analysis (Figure 1C). These data suggested that the changes in gene expression during osteoclast differentiation could positively affect the production of UDP-GlcNAc and subsequent protein O-GlcNAcylation.

Next, we assessed the global protein O-GlcNAcylation levels during osteoclast differentiation. We observed that the O-GlcNAcylation level increased gradually, peaking on day 2 (Figure 1D). Additionally, the levels of Ogt and NFATc1, a representative osteoclast differentiation marker, were also upregulated, consistent with the increase in O-GlcNAcylation (Figure 1D). Finally, we verified the role of HBP activation in osteoclast differentiation by adding glutamine and UDP-GlcNAc at an indicated concentration to the cells for 4 days. We observed that both additions caused a dramatic increase in the formation of tartrate-resistant acid phosphatase (TRAP)-specific mature osteoclasts (Figure 1E,F). These results suggested that activated HBP and increased O-GlcNAcylation play essential roles during osteoclast differentiation.

### 2.2. Inhibition of O-GlcNAcylation Interferes with Osteoclastogenesis

**Figure 2:**
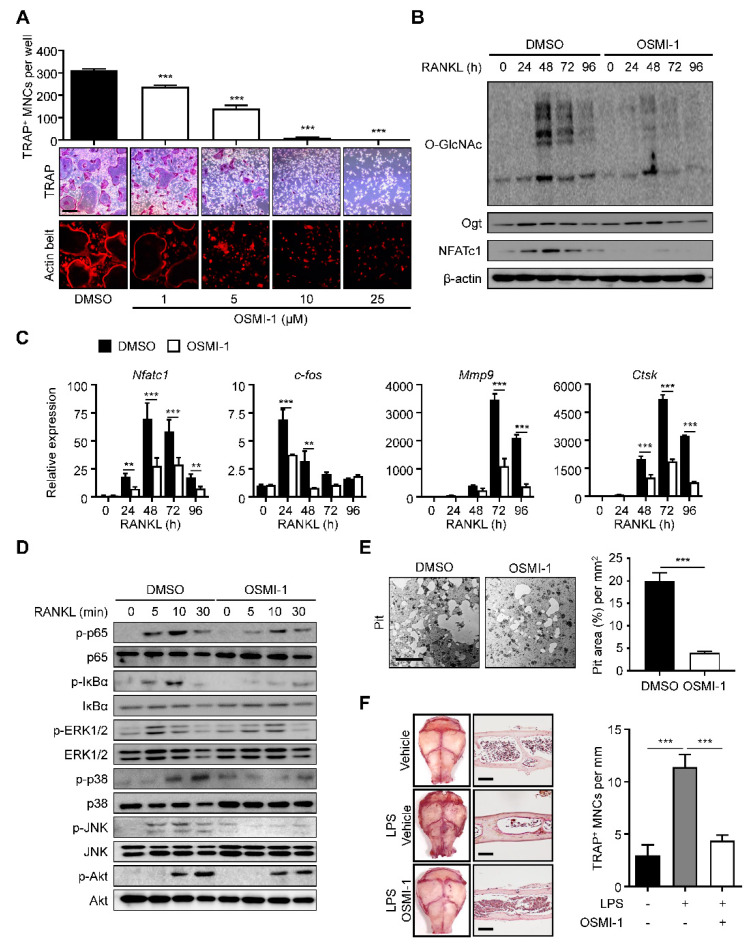
OSMI-1 inhibits RANKL-mediated osteoclastogenesis. (**A**) BMMs were incubated with M-CSF (30 ng/mL) and RANKL (100 ng/mL) in the absence or presence of OSMI-1. After 4 days, the TRAP-specific mature osteoclasts and actin belt formation were decreased by OSMI-1 in a dose-dependent manner. Scale bar, 200 μm. (**B**) The global O-GlcNAcylation, Ogt, and NFATc1 levels were measured by Western blot analysis. The global O-GlcNAcylated protein and NFATc1 expression levels were inhibited by 5 μM OSMI-1. (**C**) Relative mRNA expression levels of osteoclast markers, as measured by RT-qPCR. The mRNA ex-pression levels were decreased by 5 μM OSMI-1 during osteoclast differentiation. (**D**) Relative phosphorylation levels of the RANKL-stimulated activating signaling, as measured by Western blot analysis. (**E**) BMMs were placed on calcium-phosphate-coated plates and cultured with M-CSF (30 ng/mL) and RANKL (100 ng/mL) in the absence or presence of 5 μM OSMI-1. After 96 h, bone-resorbed areas were quantified. Scale bar, 100 μm. (**F**) Representative images of TRAP-stained surfaces and sections of calvaria tissue in a vehicle or 10 mg/kg/day OSMI-1-treated mice, into which 25 mg/kg LPS was injected. The number of TRAP-specific mature osteoclasts from stained calvaria tissue samples (left panel). Scale bar, 100 μm. Statistical data are presented as the means ± S.E.M. (** *p* < 0.01 and *** *p* < 0.001, as measured by Student’s *t*-test).

We investigated whether increased O-GlcNAcylation was critical to osteoclast differentiation and maturation. We treated the BMMs with (αR)-α-[[(1,2-dihydro-2-oxo-6-quinolinyl)sulfonyl]amino]-N-(2-furanylmethyl)-2-methoxy-N-(2-thienylmethyl)-benzeneacetamide (OSMI-I), a common Ogt inhibitor [36]. We observed that OSMI-1 successfully dose-dependently reduced the number of TRAP-positive mature osteoclasts and actin belt formation (Figure 2A). We also observed that OSMI-1 decreased the overall O-GlcNAcylation level with a significant and concomitant decline in NFATc1 levels during osteoclast differentiation (Figure 2B). The differentiation of TF, c-Fos, and NFATc1, and the late differentiation markers matrix metalloproteinase-9 (Mmp9) and cathepsin K (Ctsk) were downregulated (Figure 2C). We investigated the effect of OSMI-1 on downstream cellular signaling. We first focused on the NF-κB- and mitogen-activated protein kinase (MAPK) signaling, the early master pathways for osteoclast differentiation. Activated NF-κB and MAPK promote osteoclastogenesis by transcribing target genes, such as c-Fos and NFATc1 [26]. Although the OSMI-1 treatment hindered the phosphorylation of most downstream signaling proteins, the p65 and IκBα phosphorylation was inhibited more quickly and robustly than the molecules in the MAPK pathway (Figure 2D), suggesting that OSMI-1 suppressed RANKL-mediated differentiation signaling. Additionally, NF-kB was preferentially regulated by O-GlcNAcylation. Consistent with these results, the bone resorption activity, as determined by the size of the lacunae area in the osteologic disk, was also observed to be hindered by OSMI-1 (Figure 2E). Notably, OSMI-1 treatment effectively inhibited lipopolysaccharide (LPS)-induced formation of TRAP-positive osteoclasts in the cavarial surface compared to the mice injected with the vehicle, while OSMI-1 significantly reduced the number of TRAP-specific mature osteoclasts in the calvarial surfaces and sections (Figure 2F); therefore, OSMI-1, the Ogt inhibitor, impeded osteoclastogenesis in vitro and in vivo. 

### 2.3. RANKL Stimulation Induces O-GlcNAcylation of NF-κB p65 and NFATc1

**Figure 3:**
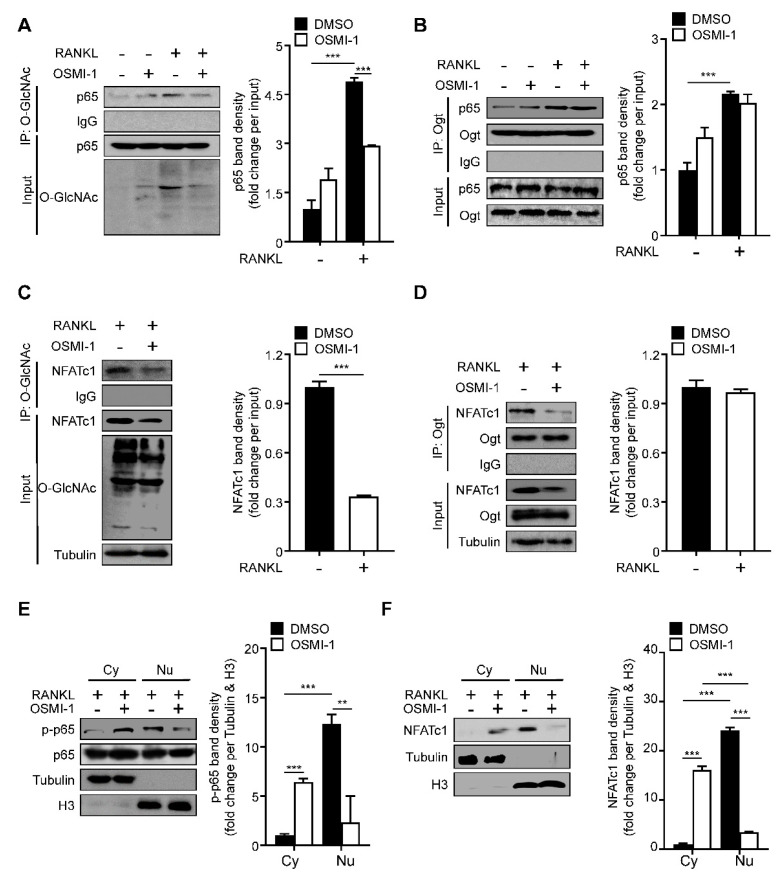
RANKL-induced p65 and NFATc1 O-GlcNAcylation are required for translocation to the nucleus. (**A**,**B**) BMMs were incubated with M-CSF (30 ng/mL) and RANKL (100 ng/mL) in the absence or presence of 5 μM OSMI-1 for 5 min. Western blot analysis of O-GlcNAc, Ogt, or p65 was performed after immunoprecipitation using O-GlcNAc or Ogt antibodies (right panel). The immunoprecipitation band density was quantified (left panel). (**C**,**D**) BMMs were incubated with M-CSF (30 ng/mL) and RANKL (100 ng/mL) in the absence or presence of 5 μM OSMI-1 for 48 h. Western blot analysis of O-GlcNAc, Ogt, or NFATc1 was performed after immunoprecipitation using O-GlcNAc or Ogt antibodies (right panel). The immunoprecipitation band density was quantified (left panel). (**E**) Cytosolic and nuclear proteins were extracted for BMMs in the absence or presence of 5 μM OSMI-1 for 5 min to determine translocation of p-p65 (right panel). The band density was quantified (left panel). (**F**) Cytosolic and nuclear proteins were extracted for BMM in the absence or presence of 5 μM OSMI-1 for 48 h to determine translocation of NFATc1 (right panel). The band density was quantified (left panel). Statistical data are presented as the means ± S.E.M. (** *p* < 0.01 and *** *p* < 0.001, as measured by Student’s *t*-test).

Next, we wanted to determine the underlying mechanism of OSMI-1. Since NF-κB p65 and NFATc1 could be O-GlcNAcylated by OGT in T and B lymphocytes [14,15], we tested whether OSMI-1 affected NF-κB p65 and NFATc1 O-GlcNAcylation during osteoclast differentiation. The O-GlcNAcylated NF-κB p65 level significantly increased (more than quadrupled) during osteoclast differentiation (Figure 3A). The binding between NF-κB p65 and Ogt was observed upon RANKL stimulation, and NF-κB p65’s binding affinity was not affected by OSMI-1 (Figure 3B). As with NF-κB p65, NFATc1 was O-GlcNAcylated upon RANKL treatment; however, OSMI-1 reduced the O-GlcNAcylated NFATc1 level (Figure 3C). Still, OSMI-1 did not affect the binding between NFATc1 and Ogt (Figure 3D). The decrease in NF-κB p65 and NFATc1 O-GlcNAcylation reduced the translocation of both TFs to the nucleus (Figure 3E,F). These data suggest that OSMI-1 inhibits the O-GlcNAcylation of NF-κB p65 and NFATc1, affecting their translocation to the nucleus and interfering with osteoclast transcription programs. 

### 2.4. Ogt Knockdown Mimics the Effects of OSMI-1

**Figure 4:**
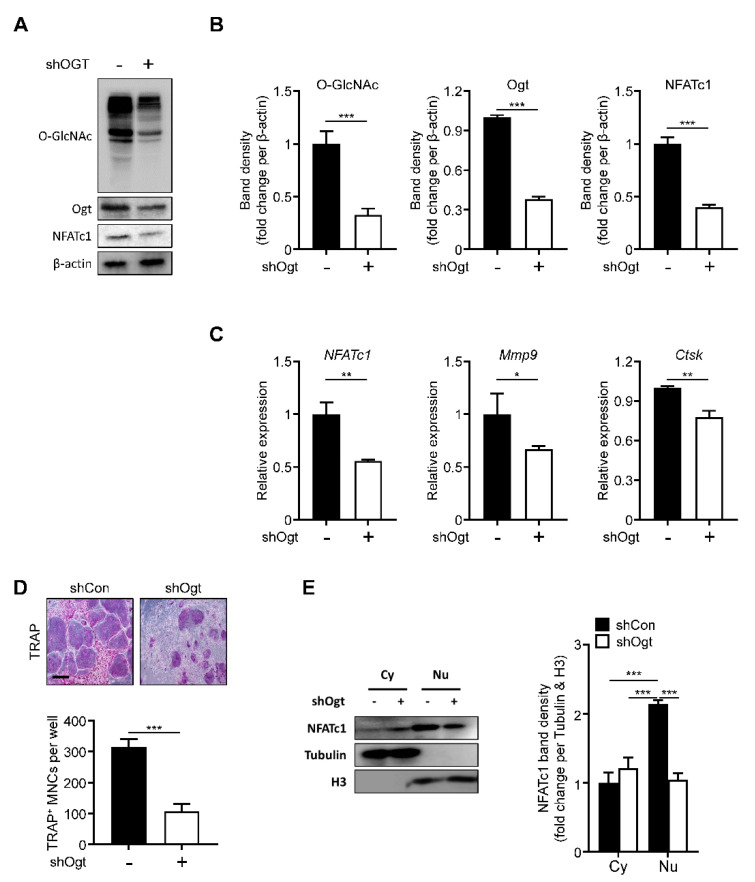
Ogt knockdown inhibits osteoclastogenesis. Infection of BMMs was performed with Ogt-targeted and control shRNA. After 48 h, the protein and mRNA expression levels were determined by (**A**) Western blot, (**B**) band density, and (**C**) RT-qPCR. (**D**) After 96 h, the TRAP-positive multinuclear cells were decreased by shOgt. Scale bar, 200 μm. (**E**) Cytosolic and nuclear proteins were extracted for infected BMMs by shOgt for 48 h to determine translocation of NFATc1. The band density was quantified (left panel). Statistical data are presented as the means ± S.E.M. (* *p* < 0.05, ** *p* < 0.01 and *** *p* < 0.001, as measured by Student’s *t*-test).

Next, we knocked down Ogt using a specific shRNA. The loss of Ogt reduced the overall level of O-GlcNAcylation. The Ogt and NFATc1 expressions were significantly decreased, comparable to the OSMI-1 treatment effect (Figure 4A,B). The NFATc1, Mmp9, and Ctsk expressions were also downregulated (Figure 4C) and TRAP-positive osteoclasts were reduced upon Ogt knockdown (Figure 4D). The translocation of the NFATc1 into the nucleus was also inhibited by the loss of Ogt (Figure 4E), showing that Ogt is a critical regulator during RANKL-mediated osteoclast differentiation.

### 2.5. Ogt Inhibition Shows a Synergistic Effect with Bone Target Therapy on Osteoclastogenesis

**Figure 5:**
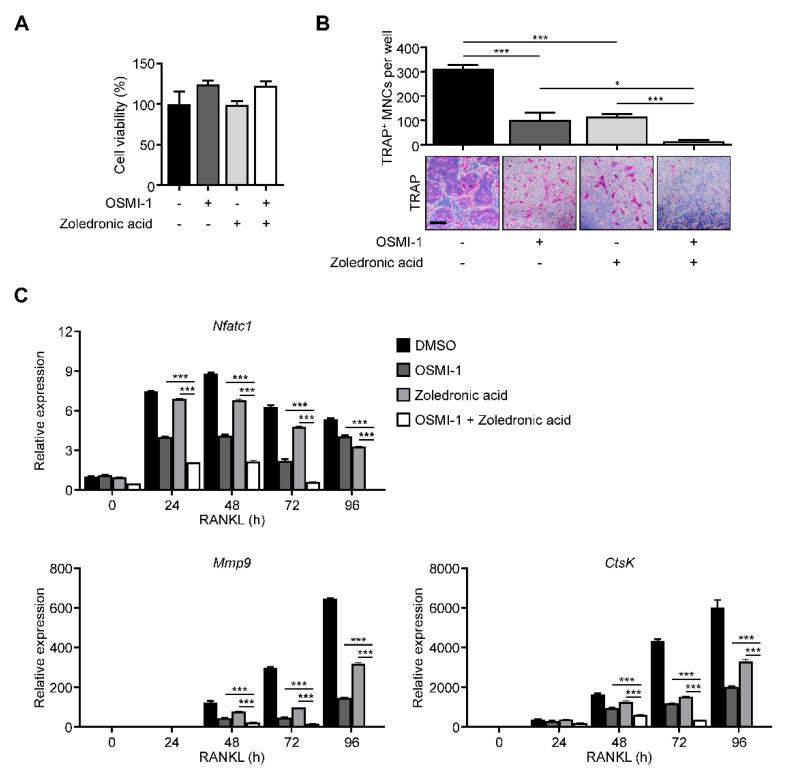
OSMI-1 enhances the effects of bone-targeted therapy on osteoclastogenesis. (**A**) Cell viability and (**B**) TRAP-specific mature osteoclasts with the drug combination of 5 μM OSMI-1 and 1 μM zoledronic acid during osteoclast differentiation, as analyzed by CCK-8 assay and TRAP staining. Scale bar, 200 μm. (**C**) Relative mRNA expression levels of osteoclast markers were measured using RT-qPCR for the drug combination of 5 μM OSMI-1 and 1 μM zoledronic acid. Statistical data are presented as the means ± S.E.M. (* *p* < 0.05 and *** *p* < 0.001, as measured by Student’s *t*-test).

To provide insights into clinical applications, we compared our method with the most popular bone target therapy drug, zoledronic acid [37]. The effect on the osteoclast differentiation was more dramatic in combination conditions without proliferation defects (Figure 5A,B). The NFATc1, Mmp9, and Ctsk expressions also followed TRAP-staining results (Figure 5C), suggesting that Ogt could be a promising combination target with current bone target therapy.

## 3. Discussion

In this study, we have demonstrated that HBP is activated, increasing global O-GlcNAcylation during RANKL-mediated osteoclast differentiation. The O-GlcNAcylation inhibition by a small-molecule inhibitor reduces osteoclast differentiation in vitro and in vivo by interfering with the translocation of TFs, NF-κB p65, and NFATc1 into the nucleus. Furthermore, Ogt deficiency confers a synergistic effect with bone target therapy on osteoclastogenesis. These findings suggest that targeting O-GlcNAcylation during osteoclast differentiation is a valuable therapeutic approach for bone-related diseases. In the near future, microcomputed tomography analysis could be desirable to determine precise bone changes, including changes in bone volume, bone mineral density, and trabecular thickness. Although the LPS-induced bone loss model is convenient for rapid analysis of osteoclast differentiation in vivo, long-term administration of OSMI-1 using an ovariectomized mouse bone loss model appears to be necessary; for this, evaluating the effects of the precise protein O-GlcNAcylation using osteoclast-specific knockouts of Ogt is worthwhile.

Cell differentiation proceeds by coordinating transcriptional networks that depend on nutrient and metabolite utilization. O-GlcNAcylation is the most nutrient-sensitive PTM because it requires UDP-GlcNAc, the end product of the HBP. The HBP generates UDP-GlcNAc by incorporating metabolites from carbohydrates, amino acids, lipids, and nucleotides. A few studies have demonstrated the impacts of HBP and protein O-GlcNAcylation on cell differentiation. HBP is favorable for adipocytes, chondrocytes, and corneal epithelial cell differentiation [38,39,40,41] but has a negative effect on embryonic cell differentiation to neuronal lineage, hematopoiesis, and skeletal myogenesis [42,43,44]. In this study, we reported the HBP is activated and that increased O-GlcNAcylation is essential for osteoclast differentiation. Furthermore, the O-GlcNAcylation of p65 and NFATc1 may be accompanied by p65 phosphorylation and p65 and NFATc1 acetylation, considering the increased transcriptional activity [14,22,45,46,47]. In the future, it will be of interest to investigate whether increased O-GlcNAcylation plays a notable role in other histone PTMs in the regulatory regions of osteoclast-specific genes and how Ogt and Oga integrate nutrients and metabolite states into chromatins with other epigenetic modifiers.

Our findings on the role of O-GlcNAcylation in osteoclasts contradict a few previous reports. For example, increased O-GlcNAcylation by an Oga inhibitor, O-(2-acetamido-2-deoxy-d-glucopyranosylidene) amino-N-phenylcarbamate (PUGNAc), or a glucosamine treatment in Raw264.7 cells inhibits osteoclast differentiation and activity [48,49,50]. This discrepancy could be due to the different cellular contexts and the off-target effects of the different treatments; however, this ironical observation reminds us of the “optimal zone” of O-GlcNAcylation [10]. Given that O-GlcNAcylation is vital for the spatiotemporal regulation of cellular processes, cells seem to have a highly sophisticated regulatory system for O-GlcNAcylation homeostasis [10]. Ogt could regulate Oga transcription and Oga activity post-translationally through O-GlcNAcylation and vice versa [51,52,53]. This mutual regulation helps to optimize the O-GlcNAcylation levels in cells; therefore, any manipulation to exit the buffering zone could interfere with the process. Nevertheless, global O-GlcNAcylation gradually increases during osteoclast differentiation and the Ogt inhibition by OSMI-1 and the *Ogt* knockout both decrease osteoclastogenesis in vitro and in vivo with a defined molecular mechanism. Thus, the O-GlcNAcylation inhibition is a better strategy than O-GlcNAcylation activation to interfere with osteoclast differentiation. 

In terms of the clinical application aspects, the effects of combination therapy with other promising bone target drugs such as denosumab should be evaluated [54]. Furthermore, many cancers can spread to the bone, and bone metastasis causes serious problems [55]. Notably, Ogt is overexpressed in many cancers and there is a positive relationship between the Ogt expression level and cancer metastatic progression [56]; therefore, the evaluation of the effects of Ogt inhibition on cell-signaling-targeting drugs such as gefitinib [57] and everolimus [58] in the context of bone metastasis could offer valuable insights that could help in developing promising therapies.

## 4. Materials and Methods

### 4.1. Isolation and Osteoclast Differentiation

C57BL/6 male mice at 5 weeks of age were purchased from Orientbio inc. (Orientbio, Gyeonggi-do, Korea). After mice were sacrificed, the whole bone marrow cells were isolated from femurs and tibias. The whole bone marrow cells were cultured in complete α-MEM medium (minimum essential medium alpha (α-MEM) with 10% fetal bovine serum (FBS; Welgene, Gyeongsangbuk-do, Korea), 2 mM L-glutamine (Gibco, Waltham, MA, USA), and 100 units/mL penicillin streptomycin (GenDEPOT, Barker, TX, USA)) for 24 h. Floating cells were cultured in complete α-MEM medium with 30 ng/mL macrophage-colony-stimulating factor (M-CSF) (ProSpec, Ness Ziona, Israel) for 72 h. After 72 h, the attached cells were used as bone-marrow-derived macrophages (BMMs) and seeded into 96-well plates at 1.5 × 10^4^ cells/well and 35 mm plate at 5.0 × 10^5^ cells/plate, then cultured in complete α-MEM medium with 30 ng/mL M-CSF and 100 ng/mL receptor activator of NF-kappaB ligand (RANKL) (ProSpec, Ness Ziona, Israel) with glutamine (Gibco, Waltham, MA, USA), UDP-GlcNAc (Sigma-Aldrich, St. Louis, MO, USA), zoledronic acid (Sigma-Aldrich, St. Louis, MO, USA), or OSMI-1 (Sigma-Aldrich, St. Louis, MO, USA) for 96 h.

### 4.2. TRAP Staining and Actin Belt Formation Assay

To identify actin belts in mature osteoclasts, the BMMs cultured in 96-well plates were fixed with 4% paraformaldehyde, pemeabilized with 0.1% Triton X-100, and stained with a rhodamine–phalloidin kit (Invitrogen, Carlsbad, CA, USA). TRAP activity in mature osteoclasts was confirmed with the leukocyte acid phosphatase kit (Sigma-Aldrich, St. Louis, MO, USA) TRAP-positive cells that contained more than three nuclei were counted as mature osteoclasts. The actin belt formation and TRAP-positive osteoclasts were scanned using a microscope (Olympus IX71; Olympus Corporation, Tokyo, Japan).

### 4.3. Cell Viability Assay 

To measure the cell viability of mature osteoclasts, BMMs were seeded into 96-well plates at 1.0 × 10^4^ cells/well in complete α-MEM medium with 30 ng/mL M-CSF and 100 ng/mL RANKL for 96 h. After 96 h, the medium was replaced with a new one composed of 90 μL of α-MEM and 10 μL of Cell Counting Kit-8 (CCK-8; Dojindo, Ningbo, China), then the cells were further incubated for 1 h. After these steps, the absorbance was measured at 450 nm, as specified in the manufacturer’s instructions.

### 4.4. Bone Resorption Assay 

To measure the resorption activity of mature osteoclasts, BMMs were seeded into Osteo Assay surface plates (Corning, Tewksbury, MA, USA) at 2.0 × 10^4^ cells/well in complete α-MEM medium with 30 ng/mL M-CSF and 100 ng/mL RANKL for 96 h. After 96 h, to remove the cells, plates were incubated with 6% sodium hypochlorite solution and washed with distilled water. Then, the washed plates were air-dried. The resorption area was measured with Adobe Photoshop CS6 (Adobe, San Jose, CA, USA).

### 4.5. Lentiviral-Mediated Gene Transduction

The shOgt constructs were purchased from Sigma-Aldrich. Letivirus production was synthesized using MISSION lentiviral packaging mix (#SHP001, Sigma-Aldrich, St. Louis, MO, USA). Lentiviral particles were produced by transfection of 293T cells using lipofectamine 2000 transfection method. The 293T cells were cultured to 60~70% confluency and letivirus production was stimulated in the cells. After 24 h, the transfection medium was changed, recombinant lentiviruses were harvested at 24 and 48 h, filtered through 0.45 µM PVDF membrane filters, concentrated using a Centricon plus-20 filter device, then aliquoted and stored at −80 °C until needed. The BMMs were seeded in 100 mm plates at 3.5 × 10^6^ cells/plate with 30 ng/mL M-CSF and infected with the filtered letivirus product. After 24 h, infected BMMs were selected with 5 μg/mL puromycin for 24 h and cultured in complete α-MEM medium with 30 ng/mL M-CSF and 100 ng/mL RANKL.

### 4.6. RNA Extraction and RT-qPCR

Total RNA was extracted using TRIzol reagent (#17061, iNtRON, Gyeonggi-do, Korea). The RNA samples were adjusted to the same concentration and reverse-transcribed using a high-capacity cDNA reverse transcription kit (#4368813, Applied Biosystems, Waltham, MA, USA). The cDNA samples were analyzed using a Cycler^®^ 480 II with LightCycler^®^ 480 SYBRGreenI master mix (#04887352001, Roche Diagnostics, Basel, Swiss), according to the manufacturer’s instructions. The RT-qPCR analysis was performed with an initial denaturation step of 5 min at 95 °C, followed by 45 cycles at 95 °C for 10 s, 60 °C for 10 s, and 72 °C for 10 s. Quantification of RNA values was determined automatically using the LightCycler^®^ 480 program (Roche Diagnostics, Basel, Switzerland). The RT-qPCR primer sequences used were as follows in the table below (Table 1).

### 4.7. Western Blot

The BMMs were lysed with RIPA buffer (50 mM Tris-HCL, pH 8.0, 150 mM NaCl, 1% Nonider P-40, 0.5% sodium deoxycholate, 0.1% SDS, and 1 mM EDTA) containing protease and phosphatase inhibitors. The lysates were centrifuged at 15,000× *g* for 10 min at 4 °C and the supernatants were transferred to a new tube. Depending on the experiment, 30 μg of whole-cell extract was separated by SDS-PAGE on 10% gels and transferred to nitrocellulose membranes. The membranes were blocked with 5% skim milk for 1 h at room temperature. After blocking, the membranes were incubated with anti-O-GlcNAc (#ab2739, 1:500Abcam, Cambridge, UK), anti-Ogt (#24083, 1:1000, Cell Signaling Technology, Danvers, MA, USA), anti-NFATc1 (SC-7294, 1:500, Santa Cruz Biotechnology, Dallas, TX, USA), anti-p65 (#8242, 1:1000, Cell Signaling Technology, Danvers, MA, USA), anti-p-p65 (#3036, 1:1000, Cell Signaling Technology, Danvers, MA, USA), anti-IκBα (#9242, 1:1000, Cell Signaling Technology, Danvers, MA, USA), anti-p-IκBα (#9246, 1:1000, Cell Signaling Technology, Danvers, MA, USA), anti-ERK1/2 (#9102, 1:1000, Cell Signaling Technology, Danvers, MA, USA), anti-p-ERK1/2 (#9106, 1:1000, Cell Signaling Technology, Danvers, MA, USA), anti-p38 (#9212, 1:1000, Cell Signaling Technology, Danvers, MA, USA), anti-p-p38 (#9215, 1:1000, Cell Signaling Technology, Danvers, MA, USA), anti-JNK (#9252, 1:1000, Cell Signaling Technology, Danvers, MA, USA), anti-p-JNK (#9251, 1:1000, Cell Signaling Technology, Danvers, MA, USA), anti-Akt (#9272, 1:1000, Cell Signaling Technology, Danvers, MA, USA), anti-p-Akt (#9275, 1:1000, Cell Signaling Technology, Danvers, MA, USA), anti-tubulin (#T9028, 1:2000, Sigma-Aldrich, St. Louis, MO, USA), anti-H3 (#ab1791, 1:2000, Abcam, Cambridge, UK), anti-β-actin (#4967, 1:2000,Cell Signaling Technology, Danvers, MA, USA), and anti-IgG (#31190, Thermo Fisher Scientific, Waltham, MA, USA) overnight at 4 °C. The membranes were washed with TBS-T and probed with an HRP-conjugated secondary antibody (#7074, 1:5000, Cell Signaling Technology, Danvers, MA, USA) in 5% skim milk for 1 h at room temperature. Following washing with TBS-T, the membranes were visualized using an enhanced chemiluminescence detection kit following exposure on an LAS-3000 image detection system (Fujifilm, Tokyo, Japan).

### 4.8. Immunoprecipitation

The osteoclasts were lysed with RIPA buffer (50 mM Tris-HCL, pH 8.0, 150 mM NaCl, 1% NP-40, 0.5% sodium deoxycholate, 0.1% SDS, and 1 mM EDTA) containing protease and phosphatase inhibitors. After centrifugation, 1 mg of the lysates was incubated with antibodies for 3 h at 4 °C, followed by subsequent incubation with A- or G-agarose beads for an additional 16 h at 4 °C. The incubated samples were washed twice with PBS and eluted with SDS sample buffer (10 mM Tris-HCl, 2% SDS, 10% glycerol, and 5% 2-mecaptoethanol). The samples were separated by SDS-PAGE, followed by Western blot analysis.

### 4.9. Cytoplasmic Extract and Nuclear Extraction

The osteoclasts were lysed with cytoplasmic extraction buffer (10 mmol/L HEPES, pH 7.9, 50 mmol/L NaCl, 0.5 M sucrose, 0.1 mmol/L EDTA, 0.5% Triton X-100, and 1 mmol/L DTT) containing protease and phosphatase inhibitors. The lysates were centrifuged at 1500× *g* for 10 min at 4 °C, then the supernatants were transferred to a new tube. The remaining pellet was lysed with RIPA buffer (CellNest, #CNR001-0100) containing protease and phosphatase inhibitors for nuclear extraction. The lysates were centrifuged at 15,000× *g* for 20 min at 4 °C, then the supernatants were transferred to a new tube. The samples were separated by SDS-PAGE, followed by Western blot analysis.

### 4.10. Calvarial Bone Destruction Assay

C57BL/6 male mice at 5 weeks of age were injected with 25 mg/kg of LPS subcutaneously over the calvaria. After 24 h, 10 mg/kg/day of OSMI-1 was injected for 6 days. Then, calvariae were separated and stained using a leukocyte acid phosphatase kit. To determine TRAP-positive cell counts, calvarial tissue samples were fixed in 4% paraformaldehyde in PBS for 6 days at 4 °C, decalcified in 14% EDTA and 9% NH4OH solution (28%) in distilled water for 7 days at room temperature, then embedded in paraffin. Serial 5 μm paraffin sections were stained for TRAP by using the leukocyte acid phosphatase kit and counterstained with hematoxylin.

### 4.11. Transcriptome Analysis

Total RNA was amplified and purified using the Target Amp-Nano Labeling Kit for the Illumina Expression Bead Chip to yield biotinylated cRNA according to the manufacturer’s instructions. Detection of array signals was carried out using Amersham fluorolink streptavidin-Cy3 according to the bead array manual. Arrays were scanned with a bead array reader and confocal scanner according to the manufacturer’s instructions. The quality of hybridization and overall chip performance were monitored manually by visual inspection of internal quality control checks and the raw scanned data. Raw data were extracted using Illumina GenomeStudio v2011.1, Gene Expression Module V.1.9.0. (Illumina, San Diego, CA, USA) Array probes were logarithm-transformed and normalized using the quantile method. The Gene Expression Omnibus accession number for this array is GSE176265.

### 4.12. Statistical Analysis

The results are expressed as the means ± standard errors of the mean. The majority of statistical comparisons were calculated using Student’s *t*-test followed by Bonferroni’s post hoc test using GraphPad Prism software version 8 (GraphPad Software, Inc., La Jolla, CA, USA). Here, *p* < 0.05 was considered to indicate a statistically significant difference.

## Figures and Tables

**Table 1:** PCR primers used in this paper.

Gene		Primer Sequences
c-fos	Forward Reverse	5′-ATCCGAAGGGAACGGAATAA-3′ 5′-TGGGCTGCCAAAATAAACTC-3′
Ctsk	Forward Reverse	5′-AGGCATTGACTCTGAAGATGCT-3′ 5′-TCCCCACAGGAATCTCTCTG-3′
Gfpt1	Forward Reverse	5′-GGAAAAGTTAAGGCACTGGATG-3′ 5′-GGGGGTGACTATTGACAGGA-3′
Mmp9	Forward Reverse	5′-TGTCATCCAGTTTGGTGTCG-3′ 5′-AATGGGCATCTCCCTGAAC-3′
Nagk	Forward Reverse	5′-TTGATTCCATCGACAACCTG-3′ 5′-CTGGCAAAATCCAGCAAACT-3′
NFATc1	Forward Reverse	5′-GCCTTTTGCGAGCAGTATCT-3′ 5′-TCATAGTGAGCCCTGTGGTG-3′
Oga	Forward Reverse	5′-CAGTACCTGGGAGAGCCAGA-3′ 5′-GTCCAAAGCACCTCAATTCC-3′
Ogt	Forward Reverse	5′-CATGCAGCTCTGGAGACAAG-3′ 5′-TGTCGATAATGCTCGATTGC-3′
Pfkl	Forward Reverse	5′-AAGAAGTAGGCTGGCACGAC-3′ 5′-GCAGGGCGTGAATACCATAG-3′
Pgm2	Forward Reverse	5′-AACAGCTCATTGCTGGAGGT-3′ 5′-GCAAAATCCCTGTGTGGTCT-3′
β-actin	Forward Reverse	5′-AAGTGTGACGTTGACATCCG-3′ 5′-GATCCACATCTGCTGGAAGG-3′

## Data Availability

The transcriptome analysis data were deposited in the NCBI*’*s Gene Expression Omnibus under GEO: GSE176265.
